# Catalytic Cleavage of the C-O Bond in 2,6-dimethoxyphenol Without External Hydrogen or Organic Solvent Using Catalytic Vanadium Metal

**DOI:** 10.3389/fchem.2020.00636

**Published:** 2020-07-28

**Authors:** Peng Yu, Xue Xie, Pengfei Tan, Wei Zhang, Zhiguo Wang, Chun Zhang, Hui Liu

**Affiliations:** ^1^College of Chemistry and Material Science, Hunan Agricultural University, Changsha, China; ^2^State Key Laboratory for Powder Metallurgy, Central South University, Changsha, China

**Keywords:** 2,6-dimethoxyphenol, vanadium metal, lignin, C-O cleavage, hydrogenolysis

## Abstract

Hydrogenolysis of the C-O bonds in lignin, which promises to be able to generate fuels and chemical feedstocks from biomass, is a particularly challenging and important area of investigation. Herein, we demonstrate a vanadium-catalyzed cleavage of a lignin model compound (2,6-dimethoxyphenol). The impact of the catalyst in the context of the temperature, reaction time, and the solvent, was examined for the cleavage of the methyl ethers in 2,6-dimethoxyphenol. In contrast to traditional catalytic transfer hydrogenolysis, which requires high pressure hydrogen gas or reductive organic molecules, such as an alcohol and formic acid, the vanadium catalyst demonstrates superior catalytic activity on the cleavage of the C-O bonds using water as a solvent. For example, the conversion of 2,6-dimethoxyphenol is 89.5% at 280°C after 48 h using distilled water. Notably, the vanadium-catalyzed cleavage of the C-O bond linkage in 2,6-dimethoxyphenol affords 3-methoxycatechol, which undergoes further cleavage to afford pyrogallol. This work is expected to provide an alternative method for the hydrogenolysis of lignin and related compounds into valuable chemicals in the absence of external hydrogen and organic solvents.

## Introduction

It provides an alternative approach to addressing renewable fuel sources and their associated environmental issues that converting renewable lignocellulosic biomass to value-added chemicals and biofuels by catalyzing (Son and Toste, 2010; Ma et al., 2018; Rinesch and Bolm, 2018; Gao et al., 2019; Liu et al., 2019). In general, the composition of the biomass, lignocellulose, is mainly composed of cellulose, lignin, and hemicellulose (Son and Toste, 2010; Yamaguchi et al., 2017, 2020; Rinesch and Bolm, 2018). Although the degradation and utilization of lignin is attractive, the development of reliable methods to access the aforementioned materials is still underdeveloped (Yamaguchi et al., 2020). For instance, most of the lignin obtained from the pulp and paper industry is either pumped into rivers as a black liquor or it is incinerated (Xu et al., 2014; Jiang et al., 2016; Wang et al., 2017). The recalcitrant and complex molecular structure of lignin accounts for the lack of enthusiasm to utilize it further (Pineda and Lee, 2016; Wang et al., 2017). Hence, an efficient and reliable method for degrading lignin by catalyzing convert to high-value products is urgently required.

Lignin is an amorphous three-dimensional hetero-polymer composed of three phenyl-propane units (sinapyl, *p*-coumaryl, and coniferyl alcohols) which are linked by relatively stable C-O and C-C bonds (Dai et al., 2016; Chen et al., 2018; Ji et al., 2018). Among them, the C-O bond is the most abundant, which accounts for 67–75% of the total linkages in lignin (Guadix-Montero and Sankar, 2018; Dong et al., 2019). Consequently, the catalytic cleavage of C-O bonds in lignin is vital to accessing high-value intermediates from the polymer; however, the selective cleavage of the C-O bonds in lignin is challenging because of the high C-O bond strength (209–348 kJ mol^−1^) (Dong et al., 2019). For the past few decades, many strategies, such as hydrogenolysis, oxidation, hydrolysis, and pyrolysis (Chu et al., 2013; Wang et al., 2013; Dai et al., 2016; Besse et al., 2017; Lin et al., 2018), have been examined for the cleavage of the C-O bond in lignin in addition to several model compounds. Among these methods, the hydrogenolysis has gained increasing attention for the degradation of lignin because of the relatively high yield and selectivity. For example, (Liu et al., 2019) investigated the hydrogenolysis of C-O bonds using Ni@ZIF-8 as catalyst, in which they demonstrated that the C-O bonds could be cleaved in the presence of a hydrogen gas under high pressure. In another variation, Jiang et al. (2019) reported the cleavage of C-O bond in lignin model compounds using a Ni/Al_2_O_3_-T catalyst with isopropanol as the hydrogen source. To this end, the benzyl phenyl ether was converted into toluene and phenol, in addition to cyclohexanol from the exhaustive reduction of phenol. Despite significant advances in the field of transfer hydrogenolytic cleavage of C-O bonds, most hydrogenolysis reactions either require high-pressure hydrogen gas or reductive organic molecules (such as alcohols and formic acid) as hydrogen donors (Hanson et al., 2010; Zhang et al., 2012; Rahimi et al., 2014; Díaz-Urrutia et al., 2016; Gomez-Monedero et al., 2017; Wang et al., 2017; Rinesch and Bolm, 2018; Kang et al., 2019; Liu et al., 2019; Yang et al., 2019). Nevertheless, hydrogen is challenging to handle and use under very high pressures and organic solvents are expensive and very often not environmentally friendly. Hence, from an environmental, economic, and practical standpoint, it is still desirable to develop new transfer hydrogenation catalytic systems that could efficiently convert lignin and the associated model compounds into valuable chemicals and thereby circumvent some of the associated limitations.

Herein, we describe the ability to employ vanadium metal as a catalyst for the cleavage of C-O bonds in 2,6-dimethoxyphenol, which is a lignin model compound, in the absence of high-pressure hydrogen gas. The impact of the reaction temperature, time, and solvent on the transfer hydrogenation activity were studied. We determined that catalytic vanadium metal has excellent activity for the hydrogenation of C-O bonds in water. Moreover, catalytic vanadium metal is effective for the transfer hydrogenation of benzyl phenyl ethers to furnish 4-benzylphenol and 2-benzylphenol.

## Materials and Methods

### Materials

All the reagents in this work were used as received without further purification.

### Experimental Procedure

All the catalytic reactions were carried out in a stainless steel autoclave reactor. The general procedure is described as follows. 2,6-Dimethoxyphenol (3 g, X mmol) and vanadium powder (0.3 g, X mmol) were weighed into an autoclave reactor and suspended in solvent 50 mL (methanol and distilled water with different volume ratio). The reactor was sealed and the atmosphere purged five times with nitrogen for the purpose of discharging air from the reactor. The catalytic reactions were conducted at a range of reaction temperatures for a specific time course. After the designated time, the autoclave was cooled to ambient temperature and depressurized carefully.

### Characterization of Catalysts

The phase structures of samples were determined by Shimadzu XRD-6000 X-ray diffractometer using Cu-Kα radiation. Tube voltage was 40 kV, Tube current was 30 mA and scan speed set to 8°/min.

### Extraction and Identification of Degradation Products

The reaction mixture was transferred into a separatory funnel, and partitioned with ethyl acetate. The organic phases were combined, dried (anhyd. CaCl_2_) filtered, and concentrated *in vacuo* using a rotary evaporator with the bath temperature set to 35°C to afford the crude material. The crude material was diluted with ethyl acetate, and then injected into organic filter membrane (0.45 μm) to permit the qualitative and quantitative GC-MS analysis of the degradation products.

## Results and Discussion

### Transfer Hydrogenation of 2,6-dimethoxyphenol at Different Reaction Time

**Figure 1:**
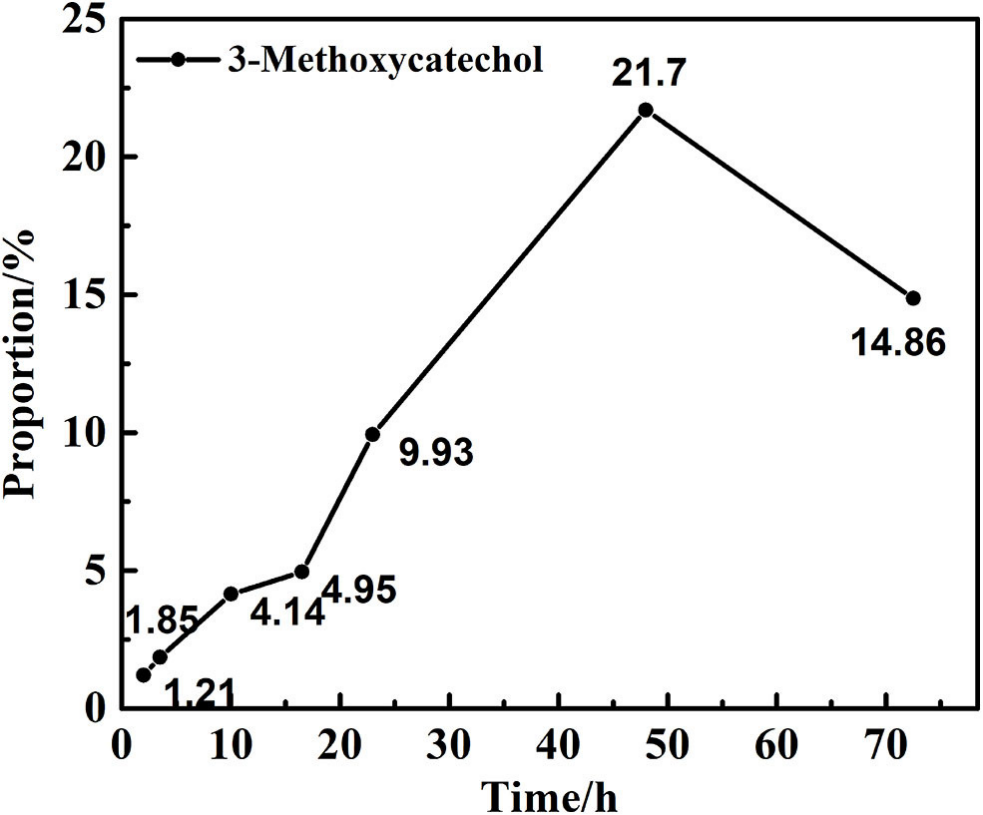
The proportion of 3-methoxycatechol in different time.

**Figure 2:**
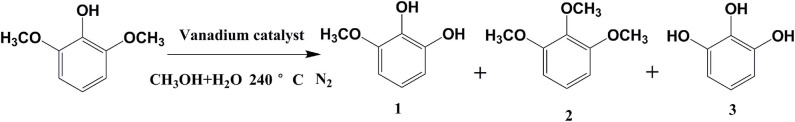
Reaction formula of 2,6-dimethoxyphenol catalyzed by vanadium under 240°C (V_H2O_:V_CH3OH_ = 4:1).

Preliminary experiments focused on the examination of the influence of the reaction time on the hydrolysis of 2,6-dimethoxyphenol with catalytic vanadium metal (Figure 1 and Table 1). Figure 1 outlines the vanadium catalyzed cleavage of the alkyl C-O bond in 2,6-dimethoxyphenol to afford 3-methoxycatechol over different time periods. Reaction formula of 2,6-dimethoxyphenol catalyzed by vanadium in different reaction time under 240°C shows in Figure 2. Vanadium could catalyze break of C-O bond in 2,6-dimethoxyphenol. Interestingly, a small quantity of 2,6-dimethoxyphenol was methylated to afford 1,2,3-trimethoxybenzene after 10 h, which is presumably the result of the activation of the C-O bond in methanol and the nucleophilic alkylation of 2,6-dimethoxyphenol. The proportion of 3-methoxycatechol increased to 22% by increasing the reaction time to 48 h, which also results in more alkylation. Interestingly, this is a rather benign method for methylation, which generally employs toxic alkylating agents. In addition, the prolonged reaction time also leads to the formation of a new degradation product, namely pyrogallol (Table 1). Hence, 3-methoxycatechol is an intermediate product, which readily undergoes further cleavage; however, extending the reaction time further to 72.5 h leads to a decrease in the amount 3-methoxycatechol to 15%, which may be ascribed to the carbonization, and the reaggregation of decomposable fragments over a long period of time.

**Table 1:** 2,6-dimethoxyphenol catalyzed by vanadium for 10 or 48 h under 240°C^a^.

**Time/h**	**Proportion/%**		
	**1**	**2**	**3**
10	4	<1	—
48	22	1	1

a *Reaction conditions: 2,6-dimethoxyphenol (3 g), vanadium catalyst (0.3 g), methanol (10 mL), distilled water (40 mL), 240°C, 1 MPa N_2_*.

### Transfer Hydrogenation of 2,6-dimethoxyphenol at Different Temperatures

**Figure 3:**
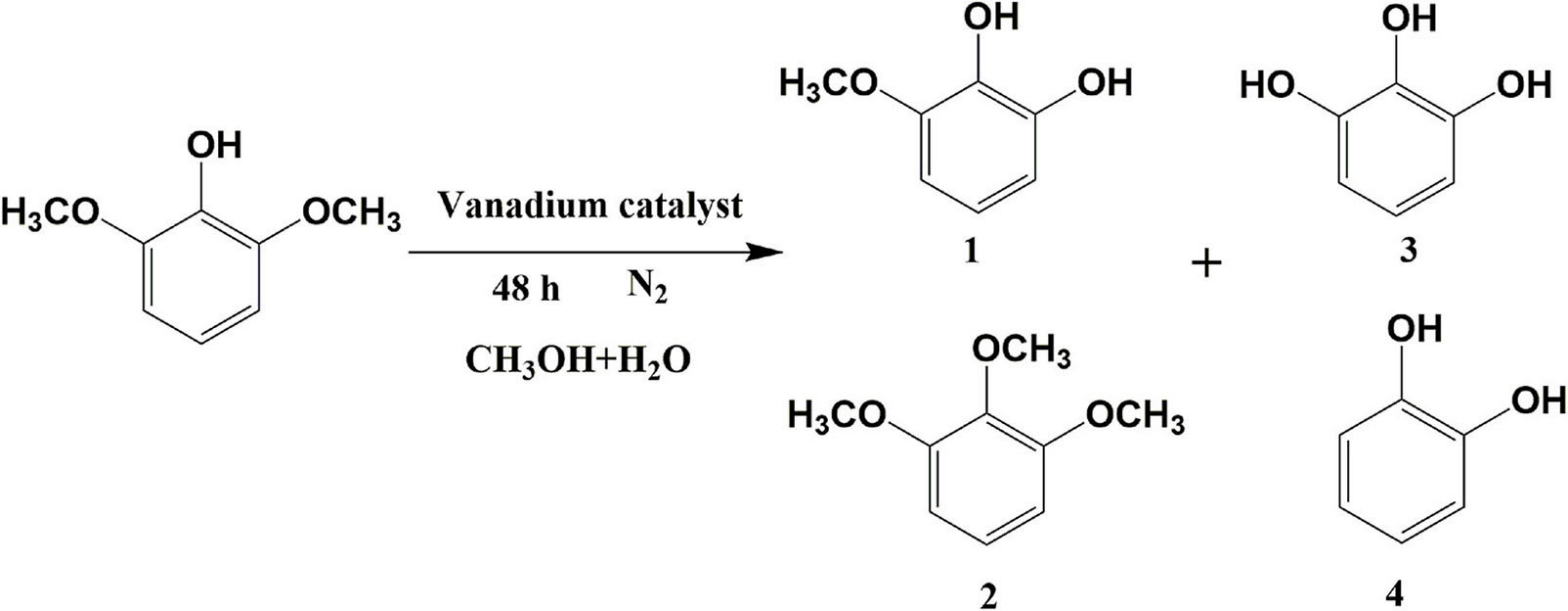
Reaction formula of 2,6-dimethoxyphenol catalyzed by vanadium under 280°C (V_H2O_:V_CH3OH_ = 4:1).

**Figure 4:**
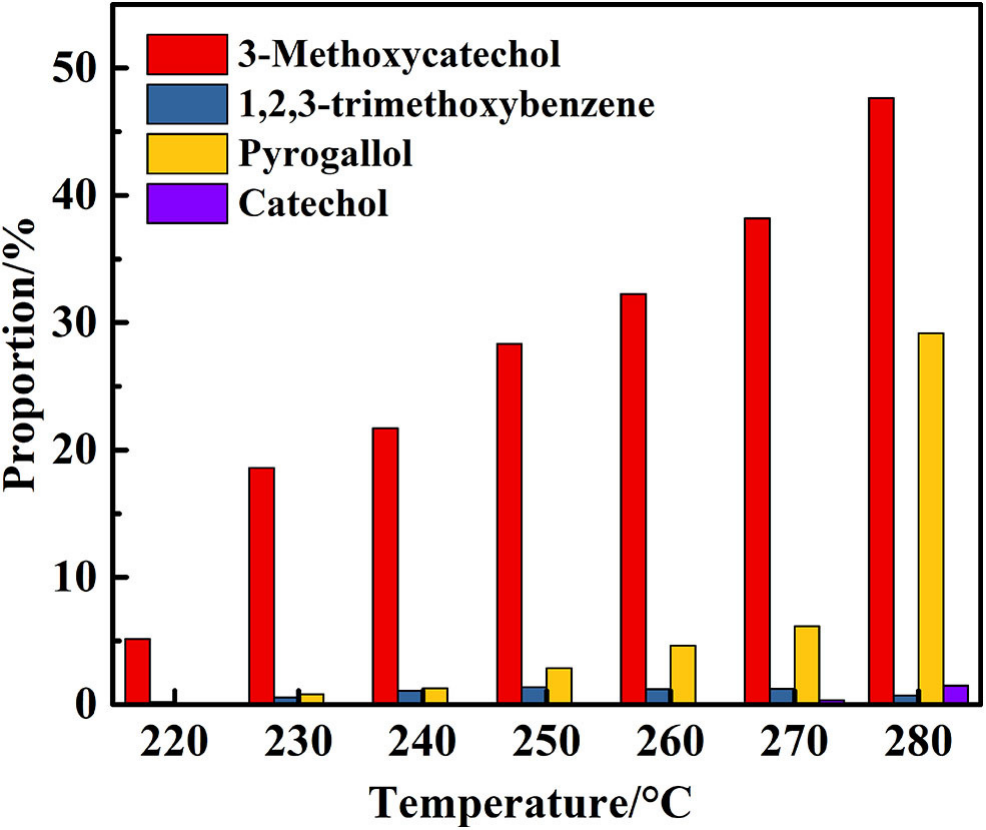
Proportion of degradation products of 2,6-dimethoxyphenol catalyzed by vanadium at different temperatures (V_H2O_:V_CH3OH_ = 4:1).

The next phase of the study examined the influence of the reaction temperature on the transfer hydrogenolysis of 2,6-dimethoxyphenol with vanadium metal as a catalyst, as outlined in Table 2. Reaction formula of 2,6-dimethoxyphenol catalyzed by vanadium under 280°C show in Figure 3. When having enough energy, besides the products of 3-methoxycatechol, 1,2,3-trimethoxybenzene, and pyrogallol, there generated new product pyrocatechol. Gratifyingly, the conversion of 2,6-dimethoxyphenol increased from 5 to 80% when the reaction temperature was increased from 220 to 280°C over 48 h. Interestingly, traces of catechol appeared at 270°C, which may be from the vanadium-catalyzed reduction of pyrogallol. Figure 4 illustrates the proportion of degradation, in which the cleavage of the 2,6-dimethoxyphenol is sensitive to the reaction temperature. Increasing the temperature to 280°C furnished 3-methoxycatechol and pyrogallol as the main products and the proportions increased to 48 and 29%, respectively. The improved yield of pyrogallol illustrates that increased temperature improves the efficiency of C-O bond cleavage in 3-methoxycatechol.

**Table 2:** The effect of reaction temperature on the reduction of 2,6-dimethoxyphenol^a^.

**Temperature/^**°**^C**	**Con/%**	**Proportion/%**			
		**1**	**2**	**3**	**4**
220	5	5	<1		
230	20	19	<1	1	
240	25	22	1	1	
250	34	28	1	3	
260	39	32	1	5	
270	47	38	1	6	<1
280	80	48	<1	29	2

a *Reaction condition: 2,6-dimethoxyphenol (3 g), vanadium catalyst (0.3 g), methanol (10 mL), distilled water (40 mL), 48 h, 1 MPa N_2_*.

**Figure 5:**
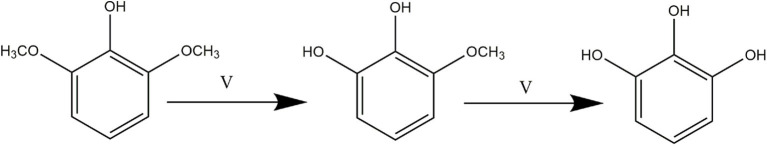
Reaction pathway of catalyzing C-O bond cleavage by vanadium.

Based on the experimental results, the reaction pathway of catalyzing cleavage of C-O bond by vanadium is proposed to follow the pathway outline in Figure 5. Initially, the vanadium-catalyzed the cleavage of C-O bond in 2,6-dimethoxyphenol affords 3-methoxycatechol, which then undergoes further cleavage to generate pyrogallol. While the origin of the formation of catechol is unclear, it could be formed from the direct aryl C-O cleavage, which means that catalyzing dehydroxylation of pyrogallol by vanadium may be feasible. Specific verification experiments on this hypothesis are outlined below.

### Catalytic Degradation of 2,6-dimethoxyphenol by Vanadium in Different Solvent

**Figure 6:**
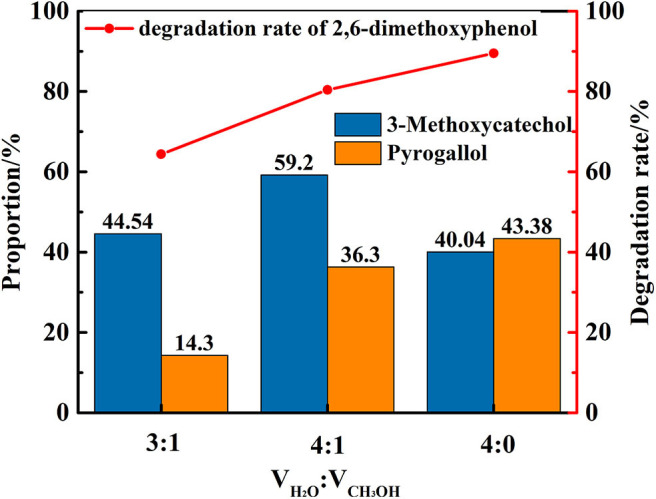
The degradation rate of 2,6-dimethoxyphenol and the proportion of main products under different distilled water/methanol volume ratio.

**Figure 7:**
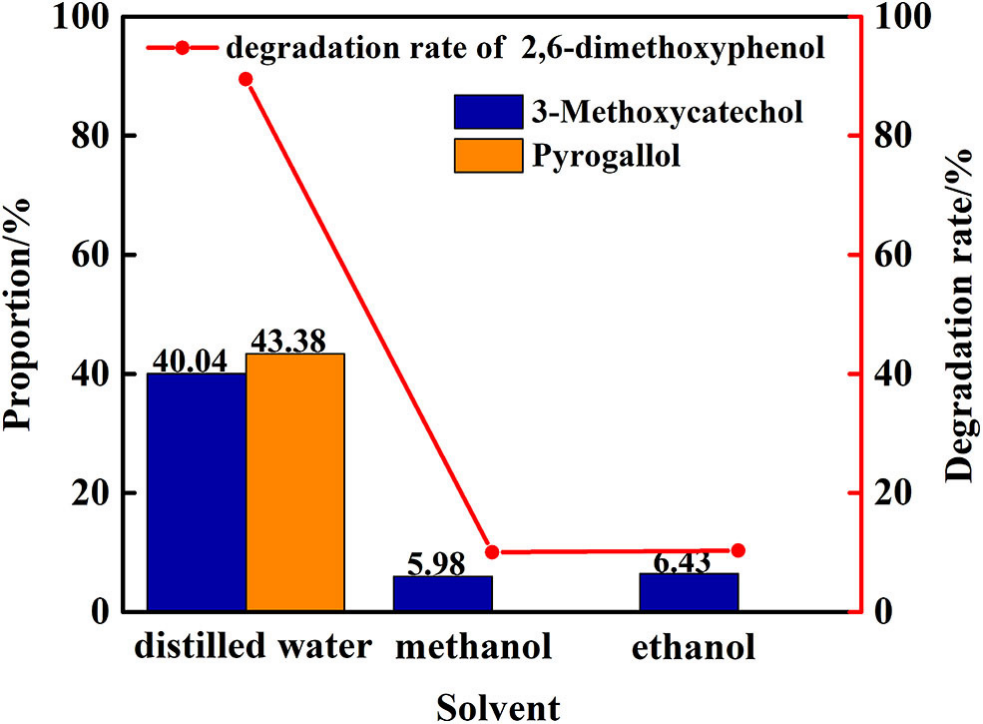
Degradation rate and main products ratio of 2,6-dimethoxyphenol in different solvents.

In the next phase of this study, the nature and impact of the solvent was examined using distilled water/methanol different volume ratio mixtures for the transfer hydrogenolysis of 2,6-dimethoxyphenol at 280°C. The proportion of main products is illustrated in Figure 6. Notably, the ratio of distilled water and methanol impacts the efficiency of the cleavage, in which only distilled water as solvent is optimal for the formation of 3-methoxycatechol and pyrogallol. Hence, the amount of water impacts the cleavage of C-O bond in 3-methoxycatechol because the proportion of pyrogallol also increased from 14 to 43%. Figure 7 delineates a comparison of the degradation 2,6-dimethoxyphenol in pure, water, and alcoholic solvents. Interestingly, the degradation in pure water is significantly more efficient than the conversions in methanol and ethanol. Moreover, pyrogallol is not produced in any of the alcoholic solvents and the proportion of 3-methoxycatechol was <7%. Hence, methanol and ethanol are significantly less efficient in generating the hydrogen necessary for C-O bond cleavage.

### Influence of Catalyst for the Transfer Hydrogenolysis of 2,6-dimethoxyphenol

**Figure 8:**
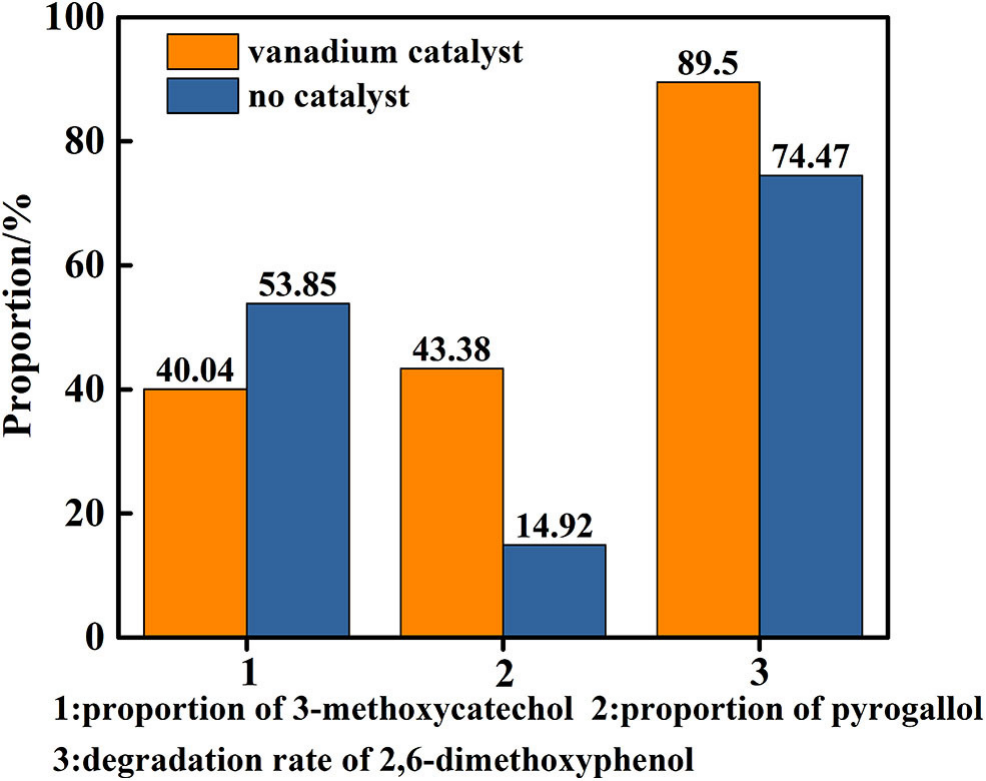
Degradation rate of 2,6-dimethoxyphenol and proportion of main products in the presence or absence of catalyst (distilled water as solvent).

**Figure 9:**
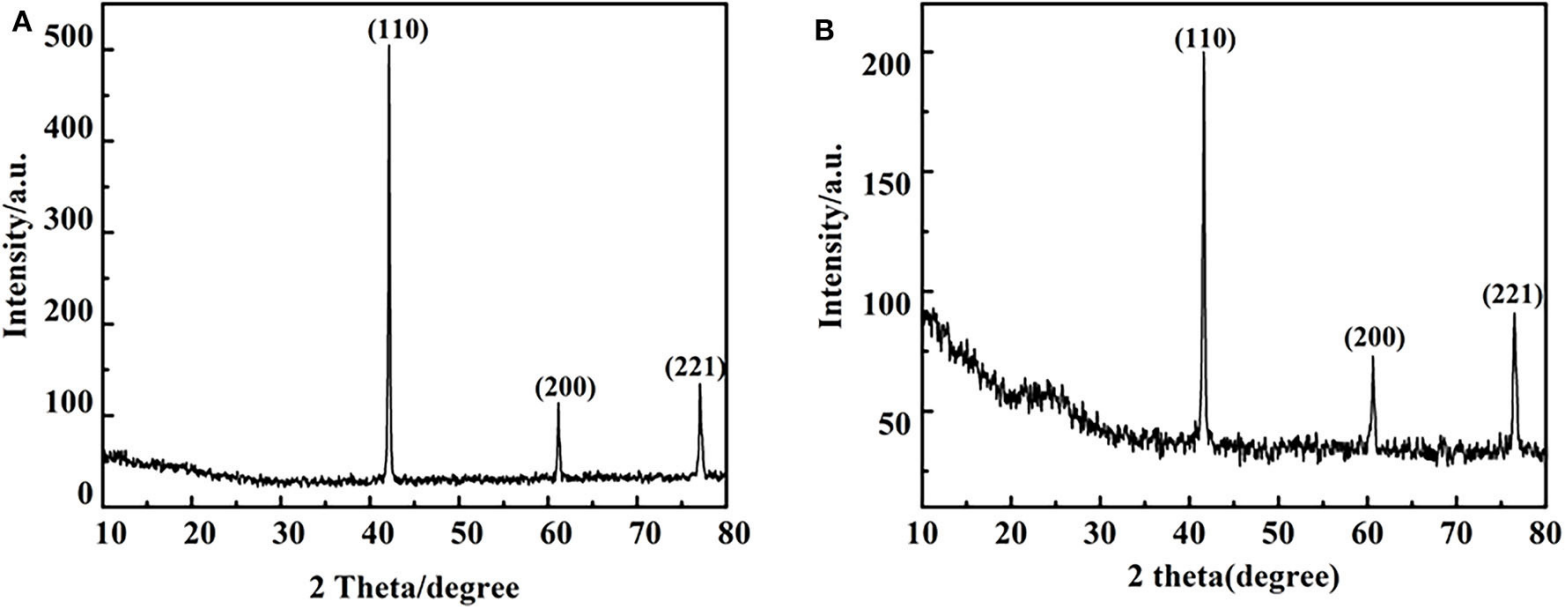
X- ray diffraction pattern before and after V catalytic reaction under 280°C: **(A)** before catalytic reaction **(B)** after catalytic reaction (distilled water as solvent).

In order to reveal the role of catalyst in the reaction, a control experiment was conducted in only distilled water as solvent at 280°C for 48 h under a nitrogen atmosphere in the presence and absence of the vanadium. The results are shown in Figure 8, which indicates the there is a background reaction because the C-O linkage in 2,6-dimethoxyphenol is cleaved in the absence of vanadium, albeit the conversion from 3-methoxycatechol to pyrogallol was not evident and the efficiency of the cleavage is lower. On the basis of this, we concluded that vanadium is necessary for the conversion of 3-methoxycatechol to pyrogallol. But as the Figure 9 shows, comparing Figures 9A,B, it is found that vanadium catalyst can maintain its basic phase structure. Differently, Figure 9B shows a another steamed bread peak around 23°, This indicates that the vanadium catalyst coking occurred at 280°C.

### Reaction Pathways of the Transfer Hydrogenolysis

**Figure 10:**
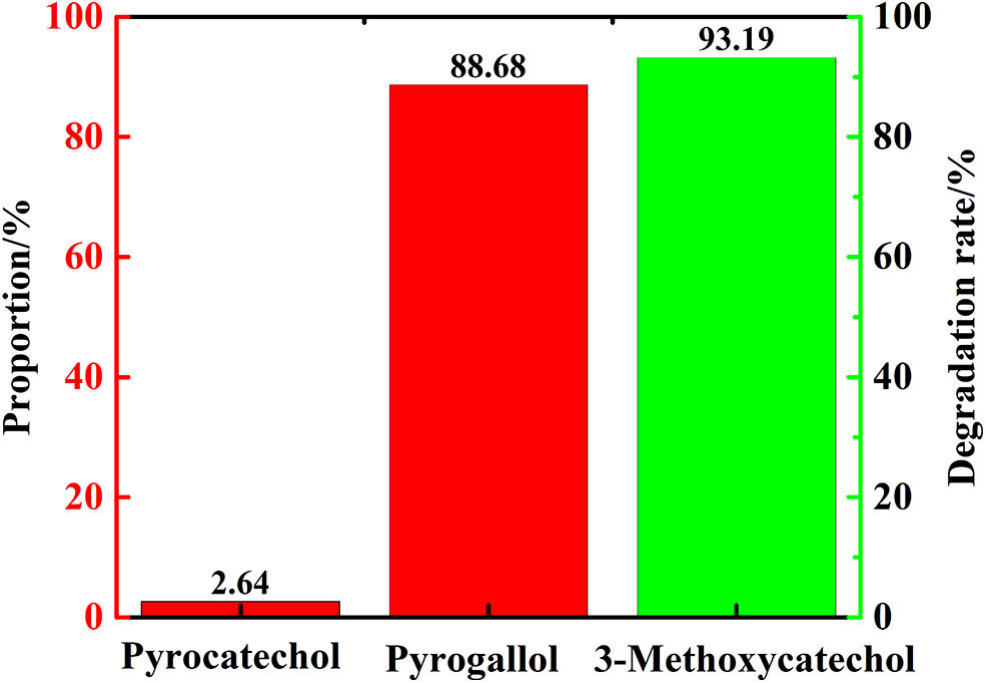
The products proportion and degradation rate of 3-methoxycatechol (distilled water as solvent).

**Figure 11:**
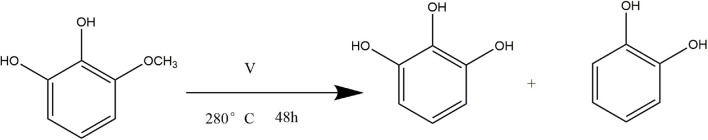
Reaction formula of vanadium catalytic cleavage of C-O bond in 3-methoxycatechol.

In order to verify the reaction path mentioned in section Transfer Hydrogenation of 2,6-Dimethoxyphenol at Different Reaction Time, that 3-methoxycatechol is intermediate product, which means vanadium can catalyze the cleavage of C-O linkage in 3-methoxycatechol to product pyrogallol. Therefore, 3-methoxycatechol was selected as the substrate to conduct experiment with only distilled water as solvent. After 48 h at 280°C, pyrogallol and catechol was formed. Figure 10 indicates the conversion of 3-methoxycatechol was 93%, which resulted in 89% of pyrogallol, in which there was only a trace of catechol formed. Hence, the vanadium catalytic cleavage of C-O bond in 3-methoxycatechol is delineated in Figure 11, in which it is confirmed that 3-methoxycatechol is the intermediate product of 2,6-dimethoxyphenol catalyzed by vanadium. Vanadium can catalyze the breaking of C-O linkage in 2,6-dimethoxycatechol or 3-methoxycatechol in distilled water, which provides a low cost and environmentally friendly process.

### The Transfer Hydrogenolysis Activity for Other Lignin Model Compounds

**Figure 12:**
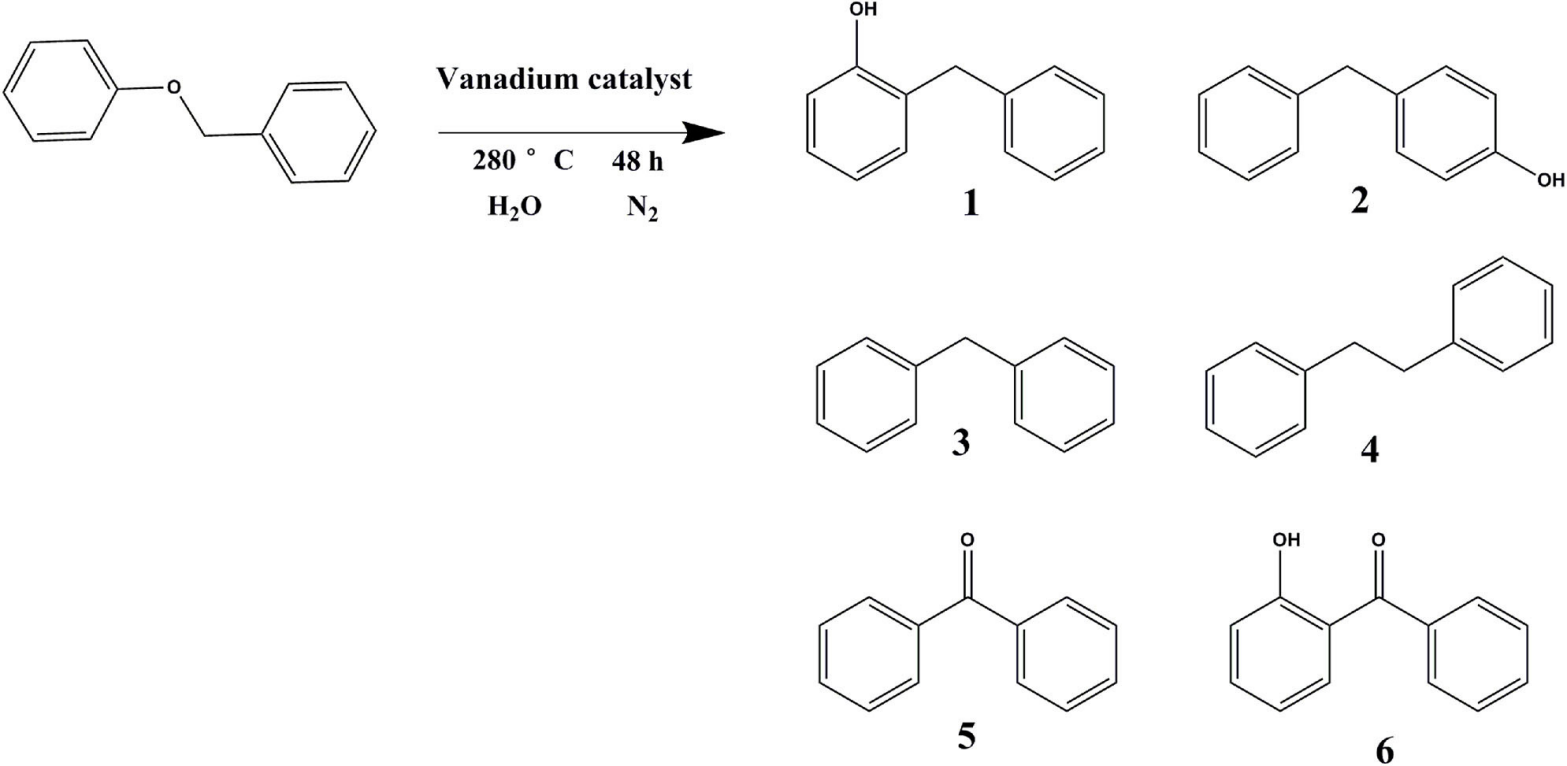
Reaction formula of benzyl phenyl ether catalyzed by vanadium under 280°C (distilled water as solvent).

In order to verify the effect of catalytic vanadium metal on the breaking of α-O-4 bond, benzyl phenyl ether was selected as the model compound. As illustrated in Table 3 and Figure 12, vanadium catalyzed the breaking of α-O-4 bond with a 98% conversion of benzyl phenyl ether. The main product was 4-benzylphenol and 2-benzylphenol. Unfortunately, the vanadium catalyst does not cleave the C-C bond, so the benzyl phenyl ether was not converted into monomer phenol. Meanwhile, the selectivity of products was not high, and many other side-products were produced. In this regard, further studies to improve the selectivity for the hydrogenation of benzyl phenyl ether and cleavage of the C-C bond to obtain monomer phenol by vanadium catalyst are required.

**Table 3:** Benzyl phenyl ether catalyzed by vanadium^a^.

**Con/%**	**Proportion/%**					
	**1**	**2**	**3**	**4**	**5**	**6**
98	42	28	<2	7	16	2

a *Reaction condition: benzyl phenyl ether (3 g), vanadium catalyst (0.3 g), distilled water (40 mL), 48 h, 280°C, 1 MPa N_2_*.

## Conclusion

In summary, vanadium metal was demonstrated to be a catalyst for the cleavage of the C-O bonds in lignin model compounds, such as 2,6-dimethoxyphenol and benzyl phenyl ether. Detailed investigations indicate that the catalyst promoted cleavage of the C-O bonds is related to reaction temperature, time, and solvent. The catalyst can efficiently catalyze the cleavage of C-O bonds in water as solvent in the absence of high-pressures of hydrogen gas and organic solvents. This work represents a promising perspective on the utilization of vanadium metal for cleaving lignin model compounds and ultimately lignin into value-added chemicals using an economical and environmentally-friendly method.

## Data Availability Statement

All datasets generated for this study are included in the article/supplementary material.

## Author Contributions

PY, HL, and WZ designed experiments. XX carried out experiments. XX and PY analyzed experimental results. ZW, CZ, and PT wrote the manuscript. All authors contributed to the article and approved the submitted version.

## Conflict of Interest

The authors declare that the research was conducted in the absence of any commercial or financial relationships that could be construed as a potential conflict of interest.
